# The use of blackcurrant pomace and erythritol to optimise the functional properties of shortbread cookies

**DOI:** 10.1038/s41598-024-54461-7

**Published:** 2024-02-15

**Authors:** Ewa Raczkowska, Aneta Wojdyło, Paulina Nowicka

**Affiliations:** 1 Department of Human Nutrition, Faculty of Biotechnology and Food Science, Wrocław University of Environmental and Life Sciences, 37 Chełmońskiego Street, 51-630 Wrocław, Poland; 2 Department of Fruit, Vegetable and Nutraceutical Plant Technology, Faculty of Biotechnology and Food Science, Wrocław University of Environmental and Life Sciences, 37 Chełmońskiego Street, 51-630 Wrocław, Poland

**Keywords:** Dietary carbohydrates, Sugar alcohols, Lifestyle modification, Nutrition

## Abstract

As a result of the production of blackcurrant juice, pomace is produced, which is a cheap, easy to further process raw material with high health benefits. The aim of the research was to develop a recipe for shortbread cookies based on blackcurrant pomace (0, 10, 30, 50%) and erythritol, and to assess their nutritional value (content of proteins, fats, sugars, dietary fibre, selected minerals and energy value), pro-health properties (antioxidant and anti-diabetic capacity) and sensory evaluation. The energy value of products with 50% of pomace sweetened with erythritol was nearly 30% lower compared to traditional cookies, while the content of dietary fibre was 10 times higher in products with the highest percentage of pomace. The antioxidant capacity and the total content of polyphenolic compounds increased with the increase in pomace content. The ability to inhibit α-amylase by shortbread cookies without pomace was about 400 times lower than those with 50% pomace. The results of the sensory evaluation showed that erythritol-sweetened cookies have more desirable characteristics compared to sucrose-sweetened cookies. Finally, it was proved that the proposed products are an excellent proposal for people struggling with food-dependent diseases, as well as being an opportunity to manage waste from the fruit industry.

## Introduction

Pomace is a by-product of the fruit and vegetable industry. Blackcurrant pomace is a source of, among others, bioactive substances, minerals and dietary fibre. The resulting large mass of fruit pomace is a serious problem for the processing industry. It is an impermanent product, and a high water content can lead to a rapid increase in microbiological contaminants. Pioneering studies on the practical use of blackcurrant residues were conducted by Mäkilä et al.^1^. The authors used blackcurrant pomace to produce healthy snacks and breakfast cereals. Blackcurrant pomace has also been used in other food products and still attracts the interest of many researchers^2,3^. The production of this type of products allowed the use of waste in the form of blackcurrant pomace, and at the same time contributed to the increase in the nutritional value of finished products, especially in terms of the content of dietary fiber and bioactivity.

One of the options for using blackcurrant pomace is baking sweet snack products. Among them, shortbread cookies are very popular, commonly eaten around the world. The main ingredients used in their production are: wheat flour, fat, sugar and egg yolks. Therefore, these products are characterised by a high energy value, and their excessive consumption may contribute to obesity and, consequently, to an increased risk of food-related diseases, including type 2 diabetes^4^. Therefore, it is necessary to modify the recipes of this type of product and give them functional features. It seems advisable to use substitutes for sucrose and wheat flour. Sugar alcohols are widely used as a substitute for sucrose, e.g. in confectionery and biscuit products, juices and chewing gums. However, most of them, when consumed in excess, can cause a laxative effect. The exception is erythritol, which is a calorie-free polyol and its sweetness is 60–70% of the sweetness of sucrose. In addition, 90% of the erythritol supplied to the body is absorbed from the small intestine. Then it is excreted in the urine, thanks to which, compared to other polyols, it is better tolerated by the human digestive system. The safety of erythritol has been confirmed in scientific studies conducted with the participation of humans and animals. In addition, the glycemic index of erythritol is zero, so its intake does not affect the increase in blood glucose and insulin. Due to the fact that erythritol has zero energy value, does not cause fluctuations in blood glucose and insulin levels, has a high digestive tolerance, and additionally does not cause tooth decay and has antioxidant properties, its use fits into the global trends of designing food recipes with targeted health-promoting properties^5^.

Therefore, an attempt was made to develop a recipe for shortbread cookies with high health-promoting values. For this purpose, eight variants of shortbread cookies were developed with a varying proportion of blackcurrant pomace (0%, 10%, 30% and 50% in relation to the flour weight) with the addition of sweeteners, sucrose and erythritol, in order to assess their nutritional value, health-promoting properties (antioxidant capacity and antidiabetic), as well as the sensory profile, as new confectionery product choices for people burdened with diet-related diseases. In order to assess the pro-health properties of the tested products, a laboratory evaluation of the energy value, nutritional value, sugar profile, content of minerals (Cu, Mg, Mn, Fe, Zn, Ca, Na and K), and quantitative and qualitative content of polyphenolic compounds was carried out. In addition, antioxidant capacity was determined using the ABTS and ORAC methods, as well as antidiabetic activity. Sensory evaluation was also performed for each cookie variants. It was assumed that it is possible to create a recipe for shortbread cookies that will be characterised by high health potential and at the same time will be acceptable to consumers.

## Materials and methods

### Reagents and chemicals

Chemicals for the determination of nutritional value were purchased from Idalia (Radom, Poland). In Merck (Darmstadt, Germany) were purchased elemental determination standards and reagents for assessing antioxidant properties (ABTS, ORAC) and in MS Spectrum (Warsaw, Poland) were purchased CRM. In Extrasynthese (Lyon, France) were purchased reagents for qualitative and quantitative polyphenolic compounds. In POL-AURA (Morąg, Poland) were purchased sugars. In Sigma-Aldrich (Steinheim, Germany) were purchased reagents for determining antidiabetic properties (basic enzyme for reaction as: α-amylase, α-glucosidase and pancreatic lipase) and dietary fibre content. Blackcurrant pomace present Certificate of the Institute of Consumer Research was purchased from a certified company GreenHerb-Kuźniar Dariusz (Łańcut, Poland). The use of plant materials in the present study complies with the international, national, and institutional guidelines and legislation. The other ingredients necessary to make the cookies were purchased from retail outlets.

### Preparation of the shortbread cookies products tested

The material studied was 8 variants of shortbread cookies—4 variants were sweetened with sucrose and 4 with erythritol. For both sucrose and erythryol-sweetened cookies, the same addition of blackcurrant pomace was used—10%, 30% and 50% by weight of flour. The raw material composition of each cookie variant is presented in Table 1.

**Table 1:** Recipe of individual variants of shortbread cookies.

Addition of blackcurrant pomace [%]	Raw material composition (%)				
	Wheat flour type 450	Blackcurrant pomace	Butter (82% fat)	Egg yolks	Sugar/erythritol
0	45.4	0.0	30.3	9.1	15.2
10	40.9	4.5	30.3	9.1	15.2
30	31.8	13.6	30.3	9.1	15.2
50	22.7	22.7	30.3	9.1	15.2

Preparation of the individual variants of the shortbread cookies consisted of four steps:

I. Wheat flour/blackcurrant pomace powder was mixed with powdered sugar (S) or erythritol (E), egg yolks and chopped butter. All ingredients were prepared using a KitchenAid model 5KPM5 (Springfield, OH, USA) for 3 min.
II. The dough was formed into a 10 cm diameter ball, wrapped in cling film and cooled for 60 min at 4 °C.
III. The dough was rolled to a thickness and circles of 0.5 and 5.0 cm diameter were cut, respectively.
IV. The shortbread cookies were baked in a convection steam oven (Rational Combi Steamer; Landsberg am Lech, Munich, Germany) for 8 min at 180 °C.

### Nutrition profile of the shortbread cookies

The energy and nutritional value of the shortbread cookies tested was determined using standard methods. Fat content was determined by the Soxhlet method^6^, proteins by the Kjeldahl method (AACC, 2000)^7^, dietary fibre by enzymatic–gravimetric method^8^. Dry matter, ash, and energy value, content were determined using commonly used AACC methods^7^.

The content of available carbohydrates in the tested shortbread cookies was calculated by subtracting from the dry matter content: the content of ash, fat, protein and dietary fibre.

### Determination of carbohydrates content

Sugar content was determined by HPLC-ELSD 1000 detector (Merck-Hitachi L-7455; Merck KGaA, Darmstadt, Germany and Polymer Laboratories Inc., Amherst, MA, USA) as method previously characterised by Wojdyło et al.^9^. The determination begins by weighing 4–5 g of the shortbread cookies sample and adding 100 mL of distilled water. The samples were then ultrasonically treated for 15 min (Sonic 6D; Polsonic, Warsaw, Poland), nextheated at 90 °C for 30 min, stirring several times during this step. Then sample was centrifuged for 10 min at 19,000×*g* (MPW-55; Warsaw, Poland) and finally supernatant was filtered using Sep-Pak C-18 cartridges (Waters Millipore, Milford, MA, USA). After purification of the samples on Sep-Pak C-18 columns, the collected fraction was again centrifuged and then filtered using PTFE hydrophilic membrane filter (0.20 mm; Millex Samplicity; Merck, Darmstadt, Niemcy). Sample injection 20 μl were analysis using some column as Prevail™ Carbohydrate ES HPLC Column-W to separate sugars (250 × 4.6 mm × 5 mm; Imtakt, Kyoto, Japonia). Analysis condition: the flow rate was 1 mL/min; temp. 30 °C; acetonitrile:water (75:25, *v/v*) was used for isocratic elution as the mobile phase. The rest parameters were used: flow nitrogen gas as 1.2 mL/min and 80 °C for the nebuliser and for the evaporation temperature. Concentrations some standards (1–10 mg/L) of sucrose, glucose, maltose and erythritol were used for calibration curves (r^2^ = 0.9998). Results are reported as the average of n = 3 replicates and expressed in g/100 g cookies.

### Determination of mineral content of the shortbread cookies

Sodium, calcium and potassium were analysis by atomic emission spectrometry (FEAS), while zinc, iron and magnesium were determined by atomic absorption spectrometry (FAAS). Determination of mineral content was made using an atomic absorption spectrometer model (Varian AA240FS; Mulgrave, VIC, Australia). About 0.5 g of each sample was weighed. To each of them was added 65% nitric acid and hydrogen peroxide in the amount of 5 and 1 mL, respectively. Samples were mineralised in a MARS 6 closed microwave system (CEM, Matthews, NC, USA) at 210 °C for 15 min. The samples were quantitatively transferred using bi-distilled water into tubes (10 mL). The accuracy of the method was confirmed by validated reference material BCR-191. The uncertainty of measurement was estimated at 5% (CEN, 2003; CEN, 2009; Food and Nutrition Institute, 2013)^10–12^.

### Quanti- and qualitative analysis of polyphenolic compounds

For quantitative and qualitative determination of phenolic compounds ultra pressure liquid chromatography connected by PDA-Q/TOF–MS and with PDA detector (Waters Corporation, Milford, MA, USA) were used. This method was previously described by Tkacz et al.^13^. For the assay, about 2.00 g of each cookie sample and about 0.50 g of blackcurrant pomace were prepared. The samples of shortbread cookies were mixed with 5–6 mL of methanol: water: ascorbic acid: acetic acid solution (3:7:2:1, *v/v*), then sonicated (Sonic-6D; Polsonic, Warsaw, Poland). Re-extraction was performed after 24 h at 4 °C, then centrifuged (10 min at 19,000×*g*; MPW-350; Warsaw, Poland). Before analysis supernatants were filtered through a hydrophilic membrane (PTFE, 0.20 μm; Millex Samplicity Filter; Merck, Germany). Detailed parameters of chromatography analysis was previously described by Wojdyło et al.^9^. Chromatography conditions: BEH C18 UPLC column (2.1 × 100 mm, 1.7 μm; Waters Corporation, Milford, MA, USA), temperature analysis was 30 °C, flow rate: 0.420 mL/min, injection:5 μL. Formic acid (2%) and 100% acetonitrile was solvent A and solvent B. PDA spectra for anthocyanins at 520 nm, flavonols at 360 nm and phenolic acids at 320 nm were measured. Quantitative evaluation was carried out for selected standards characteristic of blackcurrant fruit fractions of anthocyanins, phenolic acids, and flavonols with concentrations between 0.5 and 5 mg/mL (r^2^ ≤ 0.9998). The optimised MS parameters were: desolvation temperature 300 °C, source temperature 100 °C, desolvation and cone gas flow 300 and 40 l/h, respectively, cone and capillary voltage 30 and 2500 V, respectively. Mass scanning from 100 to 1000 m*/z* was used for MS analysis. Empower 3 and and MassLynx 4.0 ChromaLynx Application Manager (Waters Corporation, Milford, Connecticut, USA), were used for quanti- and qualitative data analysis, respectively. The results of all chromatographic analysis were present as the mean of n = 3 and expressed in mg/kg of the specified cookies variant.

### Biological activity

The analysis of the biological activity of the tested products was started by weighing about 0.5 and 2.5 g of blackcurrant pomace and each variant of shortbread cookies, respectively, and then mixed with 80% aqueous methanol with 1% HCl (9:1, *v/v*), sonicated (Sonic 6D water bath; Polsonic, Warsaw, Poland) for 15 min. Finally centrifuged (5 min, 1000×*g*).

The iron-reducing capacity of 2,2′-azino-bis (3-ethylbenzothiazoline-6-sulfonic acid) (ABTS) was determined using a Shimadzu UV-2401 PC spectrophotometer (Kyoto, Japan). The analysis was performed according to Re et al.^14^. ORAC Oxygen radical absorption capacity (ORAC) test was performed using a Shimadzu RF-5301 PC spectrofluorometer (Kyoto, Japan). The principle of analysis and its individual steps have been described earlier by Ou et al.^15^. The data are presented as the average of n = 3. The results were expressed as mM Trolox per 100 g of shortbread cookies.

Analysis of antidiabetic activity (enzyme: α-amylase, α-glucosidase and pancreatic lipase) was performed using a UV-2401 PC spectrophotometer (Shimadzu, Kyoto, Japan). The method described earlier by Nowicka et al.^16^. For each variant of cookies n = 3 replicates were performed as IC_50_. The IC_50_ expresses the amount of sample that is able to reduce the activity of a given enzyme by 50%.

### Sensory evaluation

Prior to the start of the tests, participants were informed about the purpose of the study. In the current study, 62 people participated. Each participant in the sensory evaluation was informed about the purpose and course of the study and gave written, informed consent to participate in the study. Each of them received a questionnaire to record their own sensory feelings. Sensory assessment was performed using a 9-step hedonic scale. The scoring description was as follows: 1—definitely dislike, 2—very dislike, 3—dislike, 4—slightly dislike, 5—neither like or dislike, 6—slightly like, 7—like, 8—very like, 9—definitely like. Each variant of the cookies was assessed in terms of the following characteristics: colour, taste, smell, crispness and overall acceptability^17^. The tests were conducted in a sensory laboratory, free of foreign odours, disturbing light and sound. The study was based on the guidelines of the Declaration of Helsinki^18^. The personal data of the participants of the organoleptic evaluation were coded in accordance with the guidelines of the General Regulation of the European Parliament on the Protection of Personal Data (GDPR 679/2016). No ethical approval was required for this study, because of national laws (Journal of Laws 1999, No. 47 item 480 and Journal of Laws 1997 No. 28 item 152). Participants were informed that their participation was entirely voluntary so that they could stop the analysis at any point and the responses would be anonymous.

### Statistical analysis

Statistical analysis was performed in the STATISTICA program version 13.3 (StatSoft^®^; Tulsa, OK, USA). The following analyzes were used: one-way ANOVA and Tukey’s multiple range test; p ≤ 0.05, Principal Component Analysis (PCA) and Spearman’s correlation. All data are presented as mean ± standard deviation. At least three replicates were performed for all laboratory determinations.

## Results and discussion

### Nutritional profile

The energy, nutritional value, ash and dry matter content of the tested shortbread cookies are presented in Table 2. It has been shown that the increasing proportion of blackcurrant pomace affected a significant reduction in the energy value of the tested products. This is a trend that stems directly from the energy value of the individual ingredients in the recipe. Fortification of the shortbread cookies with blackcurrant pomace, which has a significantly lower energy value than flour (132 kcal/100 g and 343 kcal/100 g respectively), resulted in the higher the proportion of pomace, the lower the energy value of the final product. In addition, the use of erythritol as a sucrose substitute significantly reduced the energy value of the products. Energy values ranged from 395.55 (EB50) to 519.72 kcal/100 g (SB0). The opposite trend was shown for dietary fibre and ash—with the increase in the addition of blackcurrant pomace, their content in the tested products increased significantly. This relationship applied to both sucrose-sweetened cookies (dietary fibre: SB0—2.79 g/100 g, SB50—25.65 g/100 g; ash: SB0—0.45 g/100 g, SB50—1.46 g/100 g) and erythritol-sweetened cookies (dietary fibre: EB0—3.17 g/100 g, EB50—27.96 g/100 g; ash: EB0—0.44 g/100 g, EB50—1.50 g/100 g). There were no significant differences in dry matter and fat content. The differences in the protein content of the different types of cookies are due to the differences in protein content between wheat flour and blackcurrant pomace (11.0 and 15.29 g/100 g, respectively). In addition, small differences in the nutritional value of cookies sweetened with sucrose and erythritol may be due to the fact that fruit pomace may be a heterogeneous material. In addition, the uncertainty of individual analytical methods should be taken into account^19^. One study involving the use of fruit pomace in cookies was that of Tarasevičienė et al.^20^. In this study, wheat flour was replaced by redcurrant pomace (10, 15 and 20%). It was shown that cookies with the highest addition of redcurrant pomace had significantly higher ash, protein and fat content compared to the other cookies variants. The addition of pomace increased the dietary fibre content of the cookies, but the relationships were not significant. In our study, as the proportion of pomace in the cookies recipe increased, the dietary fibre content increased significantly. The differences between the results of our study and those of Tarasevičienė et al. are due to the different addition of pomace (10, 30 and 50% vs 10, 15 and 20%). Another study in which blackcurrant powder was added to brittle cookies was that by Mofasser Hossain et al.^21^. It was shown that the increasing proportion of blackcurrant powder affected the reduction of sugar release during the digestion process (p ≤ 0.05). The analysis of the nutritional value of shortbread cookies with blackcurrant pomace showed their attractive nutritional profile, especially in terms of high content of dietary fiber and lower energy value. There are also studies available in the literature, based on which the nutritional value of blackcurrant pomace was determined. The content of protein (9.05–15.70 g/100 g), fat (2.50–20.20 g/100 g), dietary fibre (49.24–67.40 g/100 g) and ash (2.30–4.10 g/100 g) was assessed. However, the results of our own research and those of other authors are very diverse, depending on the analytical methods used and the method of preparing the pomace^22–25^. However, this does not change the fact that both our own research and the above-mentioned studies by other authors unequivocally show that the use of pomace in the production of shortbread cookies, as an alternative to flour, positively shapes the quality of the final product by significantly increasing the qualitative and quantitative profile of minerals, dietary fibre content and reducing the energy value.

**Table 2:** Nutritional profile, ash, dry matter in 100 g of shortbread cookies containing blackcurrant pomace.

	SB0	SB10	SB30	SB50	EB0	EB10	EB30	EB50	BP
Nutritional profile, ash and dry matter (per 100 g)									
Energy value (kcal)	519.72 ± 5.17a	506.03 ± 5.89ab	494.81 ± 14.52bc	482.84 ± 11.77c	426.26 ± 11.36d	414.62 ± 4.06de	400.49 ± 3.58e	395.55 ± 1.43e	131.56 ± 2.58
Fat (g)	31.16 ± 0.07bc	31.93 ± 0.13b	31.96 ± 0.06b	30.46 ± 0.27 cd	29.51 ± 1.68d	30.76 ± 0.14c	30.91 ± 0.01bc	33.64 ± 0.20a	nd
Protein (g)	8.41 ± 0.07ab	8.28 ± 0.13b	7.85 ± 0.00c	8.71 ± 0.00a	6.81 ± 0.24d	6.47 ± 0.25e	6.98 ± 0.00d	8.54 ± 0.25ab	15.29 ± 0.00
Total carbohydrates (g)	57.51 ± 0.05ab	55.91 ± 0.00ab	55.43 ± 0.03ab	54.69 ± 2.08a	56.66 ± 1.48ab	56.04 ± 0.37ab	55.67 ± 0.04ab	53.39 ± 0.05b	73.55 ± 0.09
Dietary fibre (g)	2.79 ± 0.14e	7.41 ± 0.40d	17.30 ± 0.21c	25.65 ± 0.56b	3.17 ± 0.03e	7.59 ± 0.24d	17.77 ± 0.23c	27.96 ± 0.32a	68.18 ± 0.06
Dry matter (g)	97.51 ± 0.03a	96.79 ± 0.02a	96.35 ± 0.02a	95.71 ± 1.44a	93.43 ± 0.03a	93.84 ± 0.10a	94.60 ± 0.01a	97.12 ± 0.04a	94.00 ± 0.06
Ash (g)	0.45 ± 0.02d	0.66 ± 0.01c	1.09 ± 0.02b	1.46 ± 0.03a	0.44 ± 0.01d	0.64 ± 0.02c	1.05 ± 0.01b	1.50 ± 0.04a	5.16 ± 0.13
Sugars content (g/100 g)									
Glucose	nd	nd	nd	0.35 ± 0.04a	nd	nd	nd	nd	0.20 ± 0.02
Sucrose	15.04 ± 1.29b	15.60 ± 0.76ab	17.66 ± 0.78a	16.67 ± 0.54ab	nd	nd	nd	nd	0.14 ± 0.01
Maltose	nd	0.15 ± 0.02a	0.25 ± 0.13a	0.31 ± 0.08a	nd	nd	nd	nd	0.06 ± 0.00
Erythritol	nd	nd	nd	nd	13.59 ± 0.30b	16.23 ± 0.11a	10.88 ± 1.42c	10.68 ± 0.16c	nd
Total sugars	15.04 ± 1.29ab	15.75 ± 0.78ab	17.91 ± 0.91a	17.32 ± 0.65a	13.59 ± 0.30bc	16.23 ± 0.11ab	10.88 ± 1.42c	10.68 ± 0.16c	0.40 ± 0.03
Mineral content (mg/100 g)									
Cu	0.19 ± 0.05d	0.48 ± 0.02c	0.83 ± 0.01b	1.07 ± 0.02a	0.20 ± 0.03d	0.45 ± 0.04c	0.75 ± 0.09b	1.18 ± 0.02a	3.13 ± 0.08
Mg	19.81 ± 0.28d	28.09 ± 0.71c	39.61 ± 0.33b	51.79 ± 0.47a	20.21 ± 0.05d	29.06 ± 1.80c	39.72 ± 0.57b	54.86 ± 3.22a	138.88 ± 3.22
Mn	0.26 ± 0.01e	0.31 ± 0.01e	0.68 ± 0.05d	1.04 ± 0.01b	0.13 ± 0.02f	0.30 ± 0.01e	0.77 ± 0.02c	1.23 ± 0.07a	4.64 ± 0.04
Fe	1.70 ± 0.03d	6.05 ± 0.80c	10.55 ± 0.40b	14.96 ± 0.28a	1.28 ± 0.16d	6.55 ± 0.15c	9.82 ± 0.47b	15.09 ± 0.31a	46.92 ± 1.71
Zn	0.74 ± 0.03de	0.82 ± 0.01bcd	0.83 ± 0.03bcd	0.91 ± 0.03b	0.66 ± 0.07e	0.78 ± 0.03 cd	0.87 ± 0.08bc	1.06 ± 0.04a	1.56 ± 0.09
Ca	42.08 ± 2.21e	72.82 ± 2.58d	112.95 ± 2.93c	149.85 ± 3.72b	40.66 ± 0.89e	79.39 ± 8.70d	109.63 ± 1.48c	163.65 ± 3.64a	285.14 ± 11.49
Na	13.13 ± 0.51c	18.67 ± 0.93ab	19.09 ± 2.05ab	16.68 ± 0.21bc	17.63 ± 0.53bc	22.79 ± 4.22a	22.79 ± 0.46a	19.33 ± 3.22ab	8.03 ± 0.55
K	68.13 ± 1.63c	90.19 ± 0.76c	120.16 ± 1.14b	150.70 ± 2.91a	70.77 ± 0.84c	87.66 ± 1.06c	120.43 ± 1.65b	162.00 ± 4.68a	481.11 ± 30.93

Values (mean of three replications) ± standard deviation followed by the same letter (a, b, c,…), within the same row, are not significantly different (p ≤ 0.05; Tukey’s test); SB: sucrose-sweetened shortbread cookies; EB: erythritol-sweetened shortbread cookies; 0, 10, 30, 50: % addition of blackcurrant pomace to flour weight; BP: blackcurrant pomace; BP were not subject to statistical analysis; nd: not detected.

#### Sugar content

As part of the research, the sugar profile of the different variants of cookies was also determined. The results of these determinations are shown in Table 2. The sugars with the highest amounts were sucrose and erythritol. This is the result of their use as sweeteners in the tested products. Total sugars ranged from 10.68 (EB50) to 17.91 g/100 g (SB30). Among sugars, glucose was also identified, which was present only in a small amount of 0.35 g/100 g in SB50 cookies, and maltose, whose content increased with the increasing proportion of pomace in sucrose-sweetened cookies, but these differences were not statistically significant. The results obtained are consistent with those of other authors who confirm a low proportion of sugars in blackcurrant pomace^22^. It is surprising that the erythritol content of the tested products is reduced along with the increase in the proportion of pomace. This may be due to the interaction of erythritol with starch and proteins and the formation of non-chemical bonds^26^. However, this mechanism is not fully understood and requires further investigation.

#### Mineral content

Table 2 also shows the content of selected minerals per 100 g of each variant of the shortbread cookies. The mineral with the highest concentration was potassium (from 68.13 mg/100 g for SB0 to 162.00 mg/100 g for EB50). The tested products also contained significant amounts of calcium (from 40.66 mg/100 for EB0 to 163.65 mg/100 g for EB50) and magnesium (from 19.81 mg/100 g for SB0 to 54.86 mg/100 g for EB50) and iron (from 1.28 mg/100 mg for EB0 to 15.09 mg/100 g for EB50). Cosmulescu et al. showed that the mineral content of currants is significantly dependent on their variety^27^. The order of mineral components according to their content in 100 g of fruit was as follows: K > Ca > Mg > Fe > Al > Na > Mn > Cu. The highest concentration was found for potassium. Depending on the variety of blackcurrant, its content ranged from 178.90 mg/100 g to 299.26 mg/100 g. In this study, the potassium content of the pomace was higher at 481.11 mg/100 g, but it is a product with a lower water content than fruit, so the content of this mineral is higher. It was observed that the mineral content of the pomace is not identical to the content of the individual cookie variants (taking into account the specific mass of the pomace used for the preparation of each cookie variant). The differences are due to the fact that the cookies, in addition to fruit pomace, also contain wheat flour and egg yolks, which also contain minerals. In addition, wheat flour contains phytic acid, which can bind, among others, iron and calcium. The process of baking cookies can also influence the mineral content. Breadaliol et al. who studied the effect of baking conditions on the mineral content of wheat bread showed that, in the case of macronutrients, as the baking temperature increases, their content in bread decreases. However, prolonging the baking time resulted in less and/or no change in the observed macronutrients. Interestingly, in the case of breads baked at 220 °C, increasing the baking time from 15 to 20 min increased the content of the macronutrients studied. This treatment may have resulted in a more intensive reduction of phytate content in the initial phase of baking, which favoured a greater availability of macronutrients. The optimal temperature for phytase activity is 55 °C, therefore during baking (especially in its initial phase) the degradation of phytic acid is probably still taking place due to the activation of phytase contained in the flour. This reduces the phytate content and thus increases the macronutrients content. In addition, it was shown that baking conditions had a significant effect only on macronutrients, while temperature and baking time were not significantly associated with micronutrients content^28,29^.

### Groups of polyphenolic compounds identified in cookies

Table 3 shows three groups of polyphenolic compounds that have been identified in shortbread cookies with blackcurrant pomace. Among anthocyanins, 4 compounds have been identified: delphinidin-3-*O*-glucoside (t_R_ = 4.012 min), delphinidin-3-*O*-rutinoside (t_R_ = 4.192 min), cyanidin-3-*O*-glucoside (t_R_ = 4.533 min), cyanidin-3-*O*-rutinoside (t_R_ = 4.721 min). These are compounds specific to blackcurrant and directly responsible for its dark colouring^30^. The application of pomace from this raw material to shortbread cookies has therefore effectively fortified the final product with these ingredients, an extremely positive trend. Anthocyanins are a fraction that is believed to have numerous health-promoting properties, including antioxidant, anti-inflammatory and phytoestrogenic capacity, anti-postprandial hyperglycemic, anti-diabetic and cardioprotective effect, neuroprotection and cognitive improvement and chemoprevention^31^.

**Table 3:** Polyphenolic compounds identified by UPLC-PDA-Q/TOF-MS in shortbread cookies containing blackcurrant pomace.

Group of polyphenols	Compound	*t*_R_ (min)	λ_max_ (nm)	[M−H]^−^ (m/z)^a^	
				MS	MS/MS
Anthocyanins	Delphinidin-3-*O*-glucoside	4.012	516	465+	303+
	Delphinidin-3-*O*-rutinoside	4.192	516	611+	303+
	Cyanidin-3-*O*-glucoside	4.533	516	449+	287+
	Cyanidin-3-*O*-rutinoside	4.721	516	595+	449/287+
Flavonols	Myricetin-3-O-rutinoside	5.701	350	625	316
	Myricetin-3-*O*-galactoside	5.794	350	479	316
	Quercetin-3-*O*-rutinoside	6.205	350	609	301
	Quercetin-3-*O*-glucoside	6.528	350	463	301
	Quercetin-3-*O*-malonylglucoside	6.736	350	549	301
	Derivative of quercetin	7.247	350	–	–
	Kaempferol-3-*O*-rutinoside	7.432	350	593	285
	Derivative of kaempferol	7.517	353	–	–
Phenolic acids	Neochlorogenic acid	1.823	323	353	191

^a^[M+H]^+^ (*m/z*) for anthocyanins were obtained in the positive ion mode.

The next group identified in cookies with addition of blackcurrant pomace is eight flavonols: myricetin-3-*O*-rutinoside (t_R_ = 5.701 min), myricetin-3-*O*-galactoside (t_R_ = 5.794 min), quercetin-3-*O*-rutinoside (t_R_ = 6.205 min), quercetin-3-*O*-glucoside (t_R_ = 6.528 min), quercetin-3-*O*-malonylglucoside (t_R_ = 6.736 min), derivative of quercetin (t_R_ = 7.247 min), kaempferol-3-*O*-rutinoside (t_R_ = 7.432 min), derivative of kaempferol (t_R_ = 7.517 min). Blackcurrant is a rich source of flavonols. The authors report that the profile of this fraction in blackcurrant can be shaped by 19 compounds^32^. Hence, the application of pomace to cookies resulted in the presence of as many as 8 of them, which qualitatively makes this group dominant. In their publications, the authors emphasize that not only the amount of polyphenol compounds, but also their specific profile and interactions of individual ingredients result in the functionality of the product. Particularly effective in the context of health-promoting properties are derivatives of quercitin and kaempferol, which are believed to have antioxidant, anticancer, anti-inflammatory, antiaggregation, vasodilatory, lipolytic, antihypertensive and antiproliferative properties on cancer cells^33,34^.

In turn, among phenolic acids, neochlorogenic acid has been identified (t_R_ = 1.823 min) at m/z = 353. It is a common compound found not only in berries, but also in stone and pome fruits, and has the following characteristics the ability to inhibit the oxidation of the LDL fraction of cholesterol and have the ability to scavenge DPPH˙, ABTS˙^+^ and OH˙ radicals, superoxide radicals^35,36^.

The study clearly shows that the application of blackcurrant pomace to shortbread cookies is reflected in the profile of bioactive compounds in the finished product. Blackcurrant fortifies cookies with three groups of polyphenolic compounds, a highly desirable phenomenon in the context of designing products with programmed health-promoting properties.

### Content of polyphenolic compounds in obtained cookies

Table 4 shows the content of polyphenolic compounds in blackcurrant pomace and the various variants of cookies. Among the polyphenolic compounds, anthocyanins were the most abundant (75.2% of total polyphenolic compounds). Flavonols (14.0%) and phenolic acids (10.8%) had a much smaller share. The total polyphenol content increased significantly with increasing addition of blackcurrant pomace (cookies with 50% pomace added had about 5 times the total polyphenol content compared to those with 10% pomace added). No statistically significant differences were shown between the variants of cookies sweetened with sucrose and erythritol with the same addition of blackcurrant pomace. The tested cookies with a varying addition of blackcurrant pomace contained the characteristic polyphenolic compounds that are present in this fruit^37^.

**Table 4:** Polyphenol concentration (mg/kg) in shortbread cookies containing blackcurrant pomace determined by UPLC-PDA-FL.

Cookie variant	Phenolic compounds													Total phenolic compounds
	Anthocyanins				Flavonols								Phenolic acids	
	Delphinidin-3-*O*-glucoside	Delphinidin-3-*O*-rutinoside	Cyanidin-3-*O*-glucoside	Cyanidin-3-*O*-rutinoside	Myricetin-3-*O*-rutinoside	Myricetin-3-*O*-galactoside	Quercetin-3-*O*-rutinoside	Quercetin-3-*O*-glucoside	Quercetin-3-*O*-malonylglucoside	Derivative of quercetin	Kaempferol-3-*O*-rutinoside	Derivative of kaempferol	n-Chlorogenic acid	
SB0	nd	nd	nd	nd	nd	nd	nd	nd	nd	nd	nd	nd	nd	nd
SB10	4.53 ± 1.23a	6.84 ± 1.01bc	nd	nd	1.31 ± 0.15a	1.74 ± 0.44a	nd	nd	0.67 ± 0.11bc	nd	nd	nd	9.48 ± 0.09bc	24.57 ± 0.31bc
SB30	9.76 ± 0.59a	18.58 ± 0.46ab	nd	22.37 ± 0.48ab	2.07 ± 0.06a	3.92 ± 0.16a	nd	nd	2.15 ± 0.33ab	nd	nd	nd	16.19 ± 0.94ab	75.03 ± 0.04ab
SB50	14.36 ± 0.40a	27.96 ± 0.89a	0.15 ± 0.02a	33.94 ± 0.14a	3.68 ± 0.10a	7.14 ± 0.49a	nd	1.74 ± 0.32a	2.63 ± 0.92ab	nd	nd	1.11 ± 0.12a	21.65 ± 0.34a	114.37 ± 1.98a
EB0	nd	nd	nd	nd	nd	nd	nd	nd	nd	nd	nd	nd	nd	nd
EB10	2.05 ± 0.51a	3.68 ± 0.38 c	nd	6.40 ± 0.04 cd	0.75 ± 0.10a	1.26 ± 0.17a	nd	nd	nd	nd	nd	nd	6.10 ± 0.18 c	20.23 ± 1.10 c
EB30	6.74 ± 0.84a	12.80 ± 0.07bc	0.10 ± 0.03ab	16.21 ± 2.39bc	1.88 ± 0.12a	3.81 ± 0.10a	nd	1.13 ± 0.05ab	2.05 ± 0.18ab	nd	nd	nd	11.25 ± 0.98bc	55.97 ± 2.18bc
EB50	13.99 ± 0.57a	26.26 ± 1.85a	0.16 ± 0.01a	31.73 ± 1.67a	4.00 ± 0.15a	7.78 ± 1.09a	nd	1.86 ± 0.05a	3.39 ± 0.49a	nd	nd	nd	18.40 ± 2.78a	107.57 ± 8.56a
BP	111.19 ± 12.16	194.81 ± 7.56	1.24 ± 0.06	243.65 ± 8.56	14.87 ± 2.52	34.09 ± 4.28	2.91 ± 0.49	8.31 ± 0.56	16.26 ± 0.70	8.07 ± 2.15	3.21 ± 1.63	6.74 ± 1.12	33.43 ± 3.39	678.79 ± 33.13

UPLC-PDA-FL—ultra performance liquid chromatography with photodiode-array, and fluorescence detectors.

Values (mean of three replications) ± standard deviation followed by the same letter (a, b, c,…), within the same row, are not significantly different (p ≤ 0.05; Tukey’s test); SB: sucrose-sweetened shortbread cookies; EB: erythritol-sweetened shortbread cookies; 0, 10, 30, 50: % addition of blackcurrant pomace to flour weight; BP: blackcurrant pomace; BP were not subject to statistical analysis; nd: not detected.

The first group of polyphenolic compounds identified in the studied variants of cookies were anthocyanins. Among them, cyanidin-3-*O*-rutinoside was predominant—from 6.40 (EB10) to 33.94 mg/kg (SB50) and delphinidin-3-*O*-rutinoside—from 3.68 (EB10) to 27.96 mg/kg (SB50). A study by Sójka and Król showed that the total anthocyanin content accounted for about 90% of all polyphenolic compounds present in blackcurrant pomace. Among them, delphinidin-3-*O*-glucoside, delphinidin-3-*O*-rutinoside, cyanidin-3-*O*-glucoside and cyanidin-3-*O*-rutinoside predominated^24^.

Flavonols are another group. Thecontent of flavonols in blackcurrant pomace was characterized by 3 dominant compounds—myricetin-3-*O*-galactoside, quercetin-3-*O*-malonylglucoside and myricetin-3-*O*-rutinoside. They constituted a total of 69.05% of this fraction in pomace. Consequently, the same compounds mainly shaped the profile of flavonols in the prepared cookies, constituting from 82.52 (SB50) to 100% (SB10, SB30, EB10) of this group of polyphenolic compounds. Sójka and Król identified mirycetine glycosides and quercetin glycosides (glucoside, rutinoside and malonylglucoside) and small amounts of kaempferol glucoside. The content of myricetin glycosides ranged from 11.5 to 30.7 mg/100 g, while quercetin glycosides ranged from 5.0 to 15.9 mg/100 g^24^.

The last group is phenolic acids, the representative of which was neochlorogenic acid. The content of this compound significantly increased with the increasing share of blackcurrant pomace in the tested shortbread cookies and ranged from 6.10 (EB10) to 21.65 mg/kg (SB50). This compound has also been identified in studies by Michalska et al.^38^. They showed in their studies that the chlorogenic acid content varied depending on the methods of obtaining powder from blackcurrant pomace and ranged from 8.4 to 71.5 mg/kg.

Among all the polyphenolic compounds, no effect of the sweetener on their content was observed in the individual variants of the cookies. However, it has been shown that the content of polyphenolic compounds in the various variants of the cookies is not identical to that of the pomace. This may be due to the fact that during the preparation of the shortbread cookies, the fruit pomace was kneaded with components characterised by a high content of proteins, lipids and polysaccharides, which may interact with polyphenol compounds and eventually modify their extractability^39^. In addition, the pomace is not a homogeneous material. Based on our own studies, the content of polyphenolic compounds commonly found in blackcurrant fruits was confirmed. Substantial content of compounds with health-promoting properties brings a number of health benefits^40^.

### Biological activities

#### Determinations of antioxidants capacity (ABTS and ORAC methods)

The antioxidant capacity of the tested products and blackcurrant pomace was assessed using ABTS and ORAC methods. The results are presented in Table 5. Both the ABTS and ORAC methods showed that the increasing addition of pomace to shortbread cookies resulted in an increase in antioxidant capacity. This applied to both sweeteners. However, a statistically significant increase was observed for the addition at the levels of 30% and 50%. The replacement of sucrose with erythritol did not significantly affect the changes in antioxidant capacity, but in most cases sucrose-sweetened cookies showed a higher antioxidant capacity compared to their erythritol-sweetened counterparts. Unlike sucrose, erythritol does not participate in Maillard reactions, so cookies with its addition may have lower antioxidant capacity. Also, the action of high temperature could generate Maillard reaction products and thus increase the antioxidant capacity^41^. It has been shown that the increase in polyphenolic compounds due to the increasing proportion of pomace was associated with higher antioxidant capacity (Tables 4 and 5). Correlation analysis was performed in order to understand broader relationships. It was shown that each type of polyphenolic compound contained in blackcurrant pomace was strongly positively correlated with the antioxidant capacity determined by both methods. For ABTS, correlations ranged from 0.8485 (neochlorogenic acid) to 0.9980 (quercetin-3-*O*-malonylglucoside), and from 0.7779 (neochlorogenic acid) to 0.9973 (delphinidin-3-*O*-rutinoside) for ORAC. Similar conclusions were reached by Mofasser Hossain et al., who showed that adding blackcurrant pomace as low as 5% to cookies significantly increased antioxidant capacity (ORAC, DPPH)^21^. It has also been shown that products sweetened with sucrose in most cases have a higher antioxidant capacity compared to cookies with erythritol. Similar conclusions were reached by Nowicka and Wojdyło, who tested, among others, the antioxidant capacity and stability of polyphenolic compounds of cherry puree prepared with the addition of various sweeteners^42^.

**Table 5:** Antioxidant (ABTS, ORAC; mmol TE/100 g) and anti-diabetic [anti-α-amylase, anti-α-glucosidase and anti-lipase; IC_50_ (mg/mL)] properties of shortbread cookies containing blackcurrant pomace.

Cookie variant	Antioxidant capacity		Enzyme inhibition		
	ABTS	ORAC	Anti-α-amylase	Anti-α-glucosidase	Anti-lipase
SB0	0.03 ± 0.00e	1.15 ± 0.07d	221.76^0.9643^*f	1122.22^0.8103^*f	16.28^0.9952^*g
SB10	0.12 ± 0.01de	1.22 ± 0.07d	87.45^0.8617^*d	1186.47^0.9970^*g	13.67^0.9747^*f
SB30	0.31 ± 0.01bc	1.55 ± 0.06c	29.54^0.9661^*b	879.21^0.7652^*d	7.44^0.9808^*d
SB50	0.37 ± 0.06ab	1.95 ± 0.02a	< 0.50^0.9268^*a	719.09^0.9729^*b	1.59^0.9085^*b
EB0	0.02 ± 0.00e	1.08 ± 0.06d	202.32^0.9899^*e	934.03^0.8812^*e	16.76^0.9820^*h
EB10	0.10 ± 0.01e	1.12 ± 0.06d	84.79^0.9745^*c	796.36^0.9833^*c	11.49^0.9399^*e
EB30	0.23 ± 0.01cd	1.57 ± 0.05bc	< 0.50^0.9606^*a	711.04^0.9956^*b	5.73^0.9233^*c
EB50	0.44 ± 0.01a	1.81 ± 0.03ab	< 0.50^0.9873^*a	601.18^0.9773^*a	0.75^0.9285^*a
BP	1.94 ± 0.10	9.56 ± 0.20	< 0.50^0.9832^*	31.75^0.9926^*	0.98^0.9360^*

All data are the mean of three measurements ± standard deviation (ABTS, ORAC). Values followed by the same letter within a column are not significantly different (p ≤ 0.05; Tukey’s test); TE: Trolox; *R value (anti-α-amylase, anti-α-glucosidase, anti-lipase); SB: sucrose-sweetened shortbread cookies; EB: erythritol-sweetened shortbread cookies; 0, 10, 30, 50: % addition of blackcurrant pomace to flour weight; BP: blackcurrant pomace; BP were not subject to statistical analysis.

#### Antidiabetic activity (analysis of α-amylase, α-glucosidase and pancreatic lipase inhibition assays)

Table 5 also provides IC_50_ values reflecting the ability of the various cookie variants to inhibit α-amylase, α-glucosidase and pancreatic lipase. In each case, the increasing proportion of blackcurrant pomace in the cookie recipe was shown to be associated with a significant reduction in the IC_50_ value and thus a more intense inhibition of enzyme activity. This relationship was found in both sucrose and erythritol-sweetened cookies. The exception was sucrose-sweetened cookies with 10% added blackcurrant pomace—for this variant the IC_50_ value was higher than for the control cookies (without pomace added). However, the difference between these cookie variants was small at only 64.25 mg/mL and only affects the ability to inhibit α-glucosidase. Perhaps it was too small a quantity of pomace to induce the inhibitory effect of the enzyme. For erythritol-sweetened cookies, the difference between EB0 and EB10 was greater at 137.67 mg/mL. In addition, it was observed that the inhibitory capacity of individual enzymes was higher for products sweetened with erythritol compared to cookies with the same addition of pomace that were sucrose-sweetened. A beneficial effect of erythritol on inhibition of α-glucosidase activity was also demonstrated by Wen et al.^43^. They investigated the kinetics of enzymes and showed that erythritol had an inhibitory effect by binding to the active place α-glucosidase. Polyphenolic compounds having the greatest effect on increasing the inhibition of α-amylase were: neochlorogenic acid (r = − 0.8200); α-glucosidase: quercetin-3-*O*-glucoside (r = − 0.9147), myricetin-3-*O*-galactoside (r = − 0.9039) and myricetin-3-*O*-rutinoside (r = − 0.9011), and pancreatic lipase neochlorogenic acid (r = − 0.8904). There are few studies in the available literature identifying the health-promoting effects of adding blackcurrant pomace to sweet snacks. The most commonly used for the production of this type of product are apple pomace^44^. However, consumption of blackcurrant and processed products reduces the glycemic and insulin response in the blood^45^. Hui et al. showed that the combination of blackcurrant powder with oat bran may be a source of bioactive compounds with antihyperglycemic effects, thus confirming the anti-amylase and antiglucosidase properties. The intensity of starch decomposition and the level of reducing sugar secretion were significantly reduced during in vitro analysis. These studies have shown that enriching carbohydrate products with blackcurrant powder may result in lower glycemia after its consumption^2,46^. This is due to the inhibition of carbohydrate breakdown by α-amylase (oral cavity) and α-glucosidase (small intestine)^47^. The smallest differences in IC_50_ values between the different cookie variants were shown for lipase. Also, McDougall et al. showed that blackcurrant extracts did not inhibit lipase activity^48^. From the data presented in Table 5, it appears that the main factor influencing the inhibition of enzyme activity is the increasing proportion of pomace in particular variants of shortbread cookies and thus of polyphenolic compounds. α-glucosidase inhibitors are particularly recommended for people with diabetes. They reduce blood glucose levels by delaying intestinal glucose absorption^43^. In addition to adding polyphenol-rich blackcurrant pomace, the substitution of sucrose for erythritol also had a significant impact. Erythritol has been shown to have antidiabetic properties by inhibiting the activity of carbohydrate-degrading enzymes, inhibiting gastric emptying, increasing intestinal passage and increasing tissue sensitivity to insulin^49^.

### Sensory evaluation

**Figure 1:**
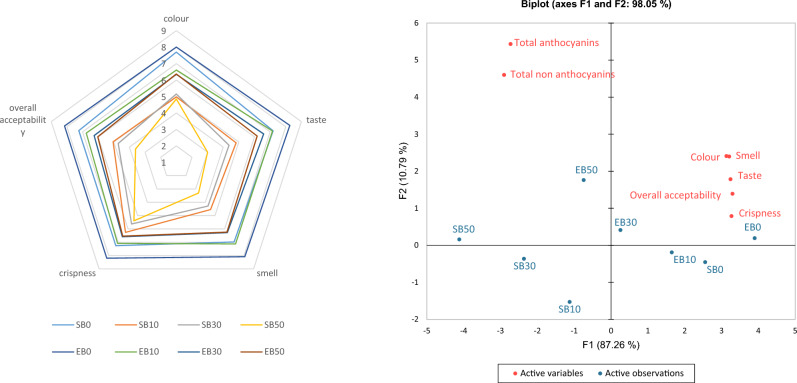
Sensory evaluation of shortbread cookies containing blackcurrant pomace; SB: sucrose-sweetened shortbread cookies with specific addition of blackcurrant pomace; EB: erythritol-sweetened shortbread cookies with specific addition of blackcurrant pomace; 0, 10, 30, 50: % addition of blackcurrant pomace to flour weight.

Figure 1 illustrates a comparison of the results of sensory evaluation of individual variants of shortbread cookies, to which two sweeteners—sucrose and erythritol were applied, as well as the influence of active variables on active observations as a result of PCA analysis. A 9-point hedonic scale was used to assess colour, taste, smell, crispness and overall acceptability. The highest overall acceptability was EB0 (8.15). For each distinguishing feature, erythritol-sweetened cookies scored higher than the corresponding sucrose-sweetened cookie variant. The lowest scores were recorded for sucrose-sweetened cookies, with 50% added blackcurrant pomace (taste—3.0, smell—3.30). For the same characteristics, the EB50 cookies scored 6.17 and 6.24 respectively. Analysis of the main components of PCA confirmed that erythritol-sweetened cookies were characterised by higher sensory acceptability than sucrose-sweetened cookies in terms of the evaluated characteristics. In addition, regardless of the addition of the sweetener, it was found that with the increasing addition of pomace, the sensory acceptability decreased among the evaluators.

A study by Górecka et al. led to different results, which showed that the addition of fruit pomace to shortbread cookies even at the level of 50% relative to the weight of flour did not negatively affect their organoleptic characteristics^50^. However, the study concerned raspberry pomace, which has a sweeter taste compared to blackcurrant pomace, which may have influenced the more favourable sensory evaluation results. Tańska et al. also used the addition of blackcurrant pomace to crisp pastry products^51^. As in the present study, it was shown that the increasing proportion of pomace affected hardness and crispness, but these were still characteristics desired by potential consumers. This study also showed that substitution of sucrose with erythritol improved sensory qualities. Lin et al. demonstrated that the use of erythritol in cookies did not affect the organoleptic evaluation results^52^. However, this examination concerned the product without the addition of pomace. In our case, the beneficial effect of erythritol on the results of the organoleptic evaluation results from the cooling effect of this polyol, which eliminated the slightly bitter aftertaste of the pomace. The feeling of cooling after consumption of products with added erythritol is also confirmed by studies by Lin et al.^52^.

### Principal component analysis (PCA)

**Figure 2:**
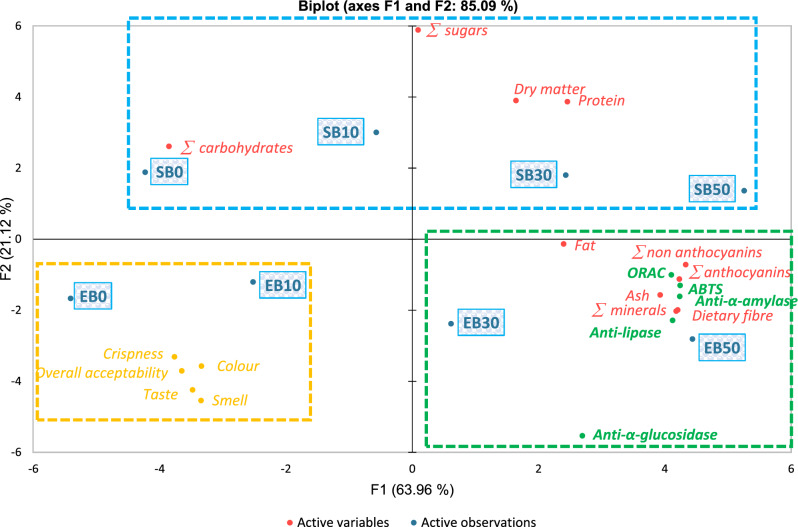
Biplot of Principal Component Analysis (PCA) representing dissimilarity relationships of chemical compounds biological activities and sensory attributes of shortbread cookies containing blackcurrant pomace.

The principal components were analysed to summarise the relationship between chemical composition, biological activity and sensory values of the tested cookies with blackcurrant pomace. The result of the PCA analysis is shown on the Biplot in Fig. 2, which shows a high interdependence of 85.09. According to the biplot, Group 1 was characterised by the correlation of the basic chemical composition (total sugar, dry matter, protein and total carbohydrates) of shortbread cookies with the addition of sucrose independently of the addition of blackcurrant pomace. All cookies: SB 0%, 10%, 30%, 50% had lower sensory acceptability than shortbread cookies EB0 and EB10. These two erythritol-sweetened products stood out in the opinion of consumers with the highest acceptability of characteristics: crispness, colour, overall acceptability, taste and smell. It should be said that all these characteristics were negatively correlated with other distinguishing features (colour: − 0.766; taste: − 0.798; smell: − 0.767; crispness − 0.863 and overall acceptability: − 0.837). Group 3 showed strong α-amylase, α-glucosidase and pancreatic lipase and antioxidant (ABTS, ORAC) properties, which significantly correlated with erythritol-sweetened shortbread cookies with the highest content of blackcurrant pomace (EB30 and EB50) (they were correlated with total anthocyanins and non-anthocyanins phenolic compounds (flavonols and phenolic acid)). In addition, the anti-glycemic and antioxidant properties of this group were also influenced by the high content of dietary fibre, ash and total minerals and, to a lesser extent, the presence of fat. Finally, it should be noted that samples of shortbread cookies other than EB30 and EB50 had the lowest anti-α-glucosidase activity, which confirms their location in different groups. However, this study has shown that 30% and 50% of blackcurrant pomace regardless of the type of sweetener provide high health potential, but with lower consumer acceptance.

## Conclusions

Blackcurrant pomace is a cheap, accessible and easy to further process waste raw material. As part of this study, a proposal of sweet snack products with high health benefits has been developed. It has been shown that the use of blackcurrant pomace and erythritol reduced the energy value of cookies (the energy value of products with 50% added pomace sweetened with erythritol was nearly 30% lower compared to traditional cookies). At the same time, the dietary fibre content increased significantly (about 10 times higher in products with 50% of pomace compared to cookies without pomace). It has also been shown that the addition of pomace has a beneficial effect on the compactness of polyphenolic compounds, antioxidant and antidiabetic activities. At the same time, the organoleptic evaluation showed that the lowest overall acceptability was for cookies with 50% added pomace, sweetened with sucrose (3.61 points). It should be noted that products with the same proportion of pomace but sweetened with erythritol obtained a much higher score—6.02 points. Research shows that it is possible to develop recipes for cookies that will be characterised by high health benefits and at the same time will be accepted by potential consumers. The use of blackcurrant pomace and the substitution of sucrose with erythritol is an excellent alternative to traditional, sweet snack products. The modifications proposed in this study are particularly important for the rapidly growing population of people with diabetes or pre-diabetic conditions and may contribute to reducing the risk of developing diabetic complications.

## Data Availability

The data supporting this study’s findings are available from the corresponding author upon reasonable request.
