# Author Correction: The use of blackcurrant pomace and erythritol to optimise the functional properties of shortbread cookies

**DOI:** 10.1038/s41598-024-55771-6

**Published:** 2024-02-29

**Authors:** Ewa Raczkowska, Aneta Wojdyło, Paulina Nowicka

**Affiliations:** 1 Department of Human Nutrition, Faculty of Biotechnology and Food Science, Wrocław University of Environmental and Life Sciences, 37 Chełmońskiego Street, 51-630 Wrocław, Poland; 2 Department of Fruit, Vegetable and Nutraceutical Plant Technology, Faculty of Biotechnology and Food Science, Wrocław University of Environmental and Life Sciences, 37 Chełmońskiego Street, 51-630 Wrocław, Poland

Correction to: *Scientific Reports* 10.1038/s41598-024-54461-7, published online 15 February 2024

The Acknowledgements section in the original version of this Article was incomplete.

“This research was funded by the National Science Centre, Poland, grant number DEC-2020/04/X/NZ9/00656 (MINIATURA 4). The publication was the result of the activity of A.W. and P.N. as the research group of “Plants4Food.””

now reads:

“This research was funded by the National Science Centre, Poland, grant number DEC-2020/04/X/NZ9/00656 (MINIATURA 4). The publication was the result of the activity of A.W. and P.N. as the research group of “Plants4Food.” The APC is financed by Wrocław University of Environment and Life Sciences.”

The original Article has been corrected.

